# A Statistical Note on Analyzing and Interpreting Individual-Level Epidemiological Data

**DOI:** 10.2188/jea.JE20140265

**Published:** 2015-04-05

**Authors:** Daisuke Yoneoka, Eiko Saito

**Affiliations:** 1 School of Advanced Sciences, Department of Statistical Science, SOKENDAI (The Graduate University for Advanced Studies), Tachikawa, Tokyo, Japan; 2 Graduate School of Medicine, The University of Tokyo, Tokyo, Japan

With the development of information technology and growing interest in collaboration with medical research, chances to analyze or interpret individual-level data have increased in the past decades, and such individual-level studies tend to be large-scale. It is important to know that even with large samples, analysis is often limited by few numbers of events and difficulty in interpreting *P*-values. In this article, we will argue several points that researchers should consider to correctly analyze and interpret individual-level data, and we will suggest some statistical methods for practitioners.

The first issue is Cox regression modeling with rare events. Parameters of interest can be estimated by the maximum likelihood method. Unfortunately, however, it is well known that the maximum likelihood estimator (MLE) becomes unreliable under “monotone likelihood” (ie, during the iterative calculation, the likelihood converges while some estimated parameters diverge to infinity).^1^ For a simple univariate case, such monotone likelihood occurs when a failed individual with the rare event has the highest or lowest value for a covariate in the risk set at each failure time, which also happens in the case of a linear combination of independent variables.^1^^,^^2^ The resultant estimates commonly produce large estimates and standard errors (SE). Although monotone likelihood is not rare and is likely to occur even with large samples, few authors tend to address this phenomenon.^1^

The same problem occurs in logistic regression models. Although the logistic regression model can always have at least one global maximum because of concavity of log-likelihood,^3^ failure of convergence (ie, monotone likelihood, also known as “complete separation” in logistic regression models) may occur when a linear combination of variables can perfectly predict the outcome.^4^^,^^5^ For example, in the simplest situation, if there is a zero cell in the 2 × 2 table formulated by dichotomous independent and dependent variables, maximum likelihood fails to converge. As a more general example, let us suppose that a large dataset has dummy variables, including five age categories and four job categories (ie, 5 × 4 = 20 categories). Under such conditions, it is reasonable to assume that everyone lacks the expected outcome for at least one category. This example produces large odds ratios and huge SEs, letting Wald-type chi-squared statistics approach zero. Although most statistical packages give an alert in such cases, researchers should pay attention when they encounter large odds ratios and confidence intervals (CIs). To diagnose whether the model is suffering from monotone likelihood, we suggest that researchers apply a simple and conventional “rule of 10 events per variable”, where each independent variable should contain at least 10 events in the use of logistic or Cox regression models.^6^

A possible solution for handling rare events would be to drop the variables suspected to cause monotone likelihood. However, we do not recommend this method because the omitted variables may have strong power to predict the outcome. Instead, it is preferable to rearrange the categories or to revert to an originally continuous variable. Another solution is to apply an exact logistic regression model, Bayesian estimation, or a penalized maximum likelihood (PML) method, which we can be easily implemented with R and SAS.^5^^,^^7^^–^^9^ Since the exact logistic regression bases the inference on exact permutational distributions of the sufficient statistics for regression coefficients of interest, performing logistic regression sometimes becomes a computer-intensive task when working with a large dataset.^10^ The Bayesian method requires a prior distribution, and the estimates are sensitive to the choice of the prior distribution. The PML method imposes penalty terms for parameters with an ordinal likelihood term. Applying Jeffrey’s prior penalty (log determinant of the Fisher information of the parameters^11^) is popular in the PML method. To handle the perfect separation problem in the case of high-dimensional data with rare events, ridge and least absolute shrinkage and selection operator (LASSO) methods are also recommended. The ridge and LASSO methods shrink regression coefficients toward zero to improve the predictive ability, but the shrinkage leads to bias in exchange for the variance (ie, bias-variance trade-off).^7^ Therefore, debiasing must be performed for shrinkage estimators, such as re-calculation of coefficients which are estimated to be non-zero, to get unbiased estimates.^7^

The second issue is the interpretation of *P*-values for statistical tests with large samples. It is common to depend only on the *P*-values to detect important exposure variables. However, over-reliance on *P*-values may lead to accepting the hypothesis with no or little significance for medical practitioners. We frequently observe articles presenting many small *P*-values such as “*P* < 0.001”. A *P*-value measures the distance between estimates (eg, odds ratios) and the null hypothesis using the unit of SE. Therefore, *P*-values derived from statistical tests, such as *t*-tests and chi-squared tests, can be formulated as a function of a sample size (ie, as the sample size increases, the CIs narrow) and the SE (ie, as SEs decrease, the CIs narrow). Large samples tend to yield smaller *P*-values (approaching zero) and larger likelihood of rejecting the null hypothesis with higher statistical power, even if the difference has no practical meaning. Statistical proof can be found elsewhere.^12^ Despite this property, many studies adhere to the conventional significance threshold of *P* < 0.05. One way to solve this problem is to stop using statistical tests and report only their point estimates and 95% CIs^13^ and let the readers interpret the significance of the findings. In using large-scale and individual-level data, researchers should interpret the results with caution by using these suggested techniques.
